# Insulin Resistance Is Inversely Associated with the Status of Vitamin D in Both Diabetic and Non-Diabetic Populations

**DOI:** 10.3390/nu13061742

**Published:** 2021-05-21

**Authors:** Shamaila Rafiq, Per Bendix Jeppesen

**Affiliations:** Department of Clinical Medicine, Aarhus University, Aarhus N, 8200 Aarhus, Denmark; per.bendix.jeppesen@clin.au.dk

**Keywords:** hypovitaminosis D, insulin resistance, fasting plasma insulin, type 2 diabetes, body mass index, vitamin D

## Abstract

Vitamin D has been implicated in the regulation of glucose metabolism and insulin resistance. We designed this study to provide evidence that insulin resistance is dependent on the concentration of vitamin D in the body. Forty observational studies of both type 2 diabetes mellitus patients and healthy subjects were included in this meta-analysis. Related articles were searched from Embase, PubMed, and Medline through January 2021. Filters for search were used to obtain more focused results. We used Comprehensive Meta-Analysis Version 3 for the construction of forest plots. RevMan software version 5.3 was used to build the risk of bias tables and summary plots. The observational studies included in this systematic review and meta-analysis showed an inverse relationship of insulin resistance with the status of vitamin D both in non-diabetic (*r* = −0.188; 95% CI = −0.141 to −0.234; *p* = 0.000) and diabetic (*r* = −0.255; 95% CI = −0.392 to −0.107, *p* = 0.001) populations. From the meta-analysis we concluded that hypovitaminosis D is related to increased levels of insulin resistance in both type 2 diabetes patients and the healthy population all over the world.

## 1. Introduction

Insulin resistance and type 2 diabetes mellitus (T2D) are among the greatest challenges of this time. Obesity is one of the major risk factors for the spread of these diseases [1]. Insulin, the glucose lowering hormone, has an important role in the adipose tissues, liver, and skeletal muscles. After binding to its receptors in the cell membrane, the insulin starts metabolic reactions, e.g., it stores glucose in the skeletal muscles and liver, initiates glucose use in the skeletal muscles, and is involved in the regulation of genes related to lipid synthesis and glucose transport. Insulin also functions to suppress lipolysis in the liver, reducing the concentration of acetyl-CoA, thus decreasing pyruvate carboxylase activity. A decrease in pyruvate carboxylase and glycerol production helps insulin reduce gluconeogenesis [2,3]. A higher insulin level in the blood to maintain a normal status of glucose defines insulin resistance. Insulin resistance is found to be the culprit for a number of diseases such as pre-diabetes, non-alcoholic fatty liver (NAFL), and polycystic ovaries [4,5,6]. Continuous high insulin requirement exhausts the beta cells of the islets of Langerhans, resulting in the obvious progression of type 2 diabetes. Hypovitaminosis D is considered to be related to the development of T2D, as evident from a number of epidemiological studies [7,8,9]. Deficiency of vitamin D is also potentially linked with non-alcoholic fatty liver disease, cardiovascular disease, and overall mortality risk [10,11,12]. Vitamin D is a fat-soluble prohormone steroid that has endocrine, paracrine, and autocrine functions [13]. Studies showed that deficiency in vitamin D develops insulin resistance, which in turn promotes obesity and type 2 diabetes [14]. The 1α-hydroxylase enzyme required for the conversion of 25 (OH) vitamin D into its functionally active form 1,25 (OH)2 vitamin D and vitamin D receptor (VDR) are found in the beta cells, showing its role in the homeostasis of insulin production [15]. The progression of diabetes is slowed with vitamin D supplementation in animal models of diabetes. Moreover, a high risk of type 2 diabetes and an intensive hyperglycemia have been observed for carbohydrate consumption under hypovitaminosis D conditions [16,17]. A strong link has been found between vitamin D status and insulin response in tissues in non-diabetic subjects [18]. The evidence of vitamin D correlation with insulin resistance is continuously increasing all over the world, showing an inverse relationship between them, which is consistent with our hypothesis [19,20].

The goal of this review was to reveal the relationship of vitamin D status and fasting plasma insulin as a measure of insulin resistance in previous diabetic and non-diabetic observational studies. The prospective relationship of vitamin D levels and insulin resistance was examined in this study using a forest plot. Vitamin D status is also affected by sun; therefore, latitude can have an effect on this relationship. Other factors that can affect this association are the method of vitamin D determination and BMI of the selected population. To identify the influence of these parameters on the relationship between vitamin D and insulin resistance, we performed meta-regression analysis.

## 2. Materials and Methods

Three databases (Embase, Medline, and PubMed) were searched for this review article to find appropriate observational studies through to January 2021. The keywords used were: “cholecalciferol”, “25 (OH) vitamin D”, “25 (OH) D”, “vitamin D3”, “vitamin D”, in combination with “fasting plasma insulin”, “HBA1C”, “homeostasis model assessment of insulin resistance”, “fasting plasma glucose”, “type 2 diabetes”, “T2D”, “adiposity”, and “abdominal obesity”. The search for the keywords was performed both as free keywords and in combination with EMTREE in Embase, and Medical Subject Heading (MeSH) in PubMed. The studies selected showed the relationship between vitamin D (25-hydroxy vitamin D) and fasting plasma insulin. The selection criteria included studies conducted on human beings of more than 18 years of age, written in English. Editorials, commentaries, and reports were not included in this study. The articles were also searched by other sources in addition to systematic search for more references. If the articles lacked necessary information on moderators or estimates, the authors were contacted.

### Statistical Analysis and Outcome Measures

The aggregate effect measure was extracted and pooled for meta-analysis as a correlation coefficient. We used the random effect model to compute the forest plot as the summary measure for the outcome. Studies were collected from a range of populations in different regions of the world with different ethnicities, cultures, and customs, since the biological effect of vitamin D varies with location. The estimates of consistency and reliability were tested by I^2^ and τ^2^, respectively, where I^2^ defines total heterogeneity as percentage among included studies.

Grades of Recommendation Assessment Development and Evaluation (GRADE) was used for quality assessment of the articles. The factors that determined the quality of the study were: 1. indirectness (compromised generalizability of results); 2. inconsistency (unexplained heterogeneity between studies); 3. publication bias (small number of participants); 4. imprecision (confidence intervals too long). Comprehensive Meta-Analysis Version 3 (Biostat, Inc., Englewood, NJ, USA) was used to perform meta-analysis. Meta-regression was performed (Comprehensive Meta-Analysis Version 3, Biostat, Inc., Englewood, NJ, USA) to determine the sources of bias. The risk of bias (ROB) analysis was performed using Review Manager 5.3.

## 3. Results

**Figure 1:**
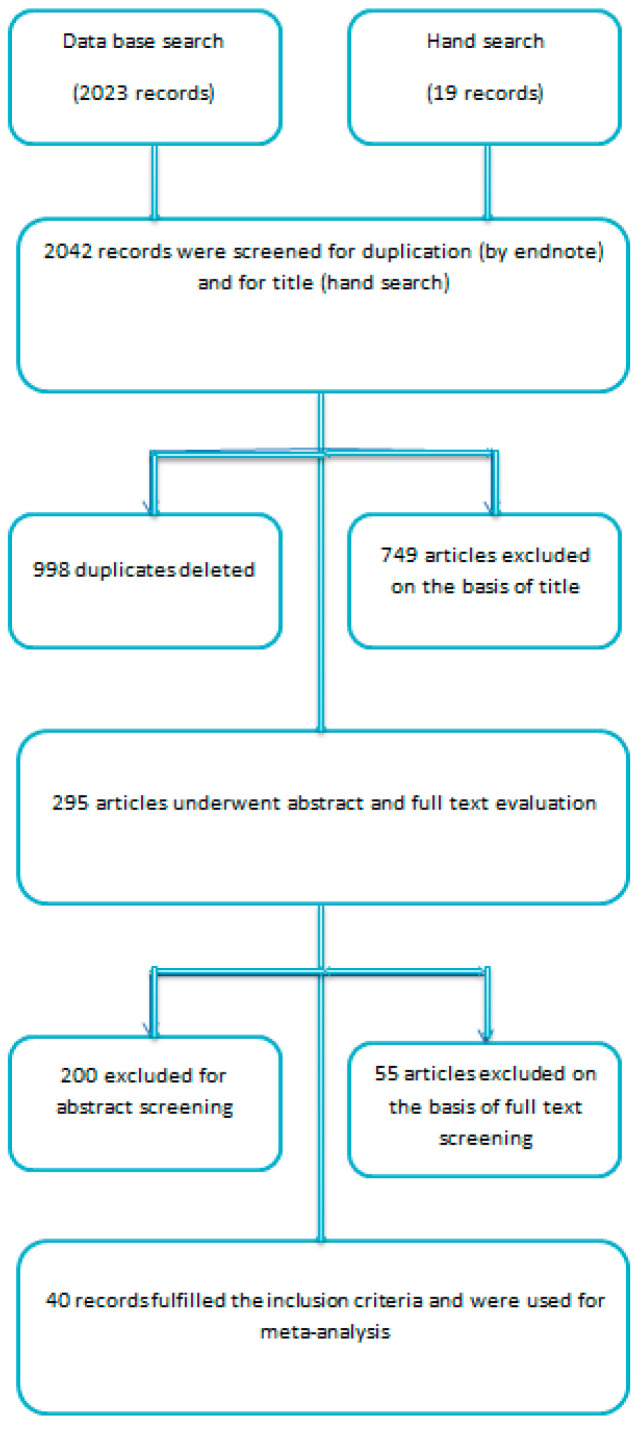
Flow chart for the literature review and selection of studies.

A total of 2023 studies were identified electronically (Pubmed, Embase, and Medline). Nineteen references were recognized by other means. Endnote software was used to screen the duplicate entries and 998 entries were discarded. A total of 749 studies were excluded on the basis of title. The rest underwent abstract and full text evaluation. The abstract evaluation discarded 200 articles and systematic assessment of full text rejected 55 articles. Forty articles fulfilled the inclusion criteria and were finally selected as eligible to be used in the meta-analysis (Figure 1).

### 3.1. Excluded Studies on the Basis of Full Text Evaluation

Eleven articles were excluded as data were not compatible with our outcome measure of correlation coefficient [14,21,22,23,24,25,26,27,28,29,30]. Twenty-one studies were selected for exclusion because their study design did not match our study design [31,32,33,34,35,36,37,38,39,40,41,42,43,44,45,46,47,48,49,50,51]. Sixteen articles were rejected because the outcome measure was calculated for a mixed population, i.e., for both diabetic and non-diabetic subjects [52,53,54,55,56,57,58,59,60,61,62,63,64,65,66,67]. One study was excluded because the number of subjects in each vitamin D quartile was not mentioned [68]. For seven references, full-length articles were not accessible [69,70,71,72,73,74,75].

### 3.2. Included Studies

#### 3.2.1. Meta-Analysis and Meta-Regression for Non-Diabetes Patient Studies

Thirty-five studies included in this meta-analysis were collected through to January 2021. The participants of all studies were at least 18 years old. Twelve studies determined vitamin D concentration by radioimmunoassay (RIA), five by enzyme-linked immunosorbent assay, eight by chemiluminescence assay (CLIA), three by electrochemiluminescence assay (ECLIA), four by liquid chromatography-mass spectrometry (LC-MS), one by high-performance liquid chromatography (HPLC), and two studies did not mention the method of determination. The articles selected were from all over the world and from different ethnicities.

**Figure 2:**
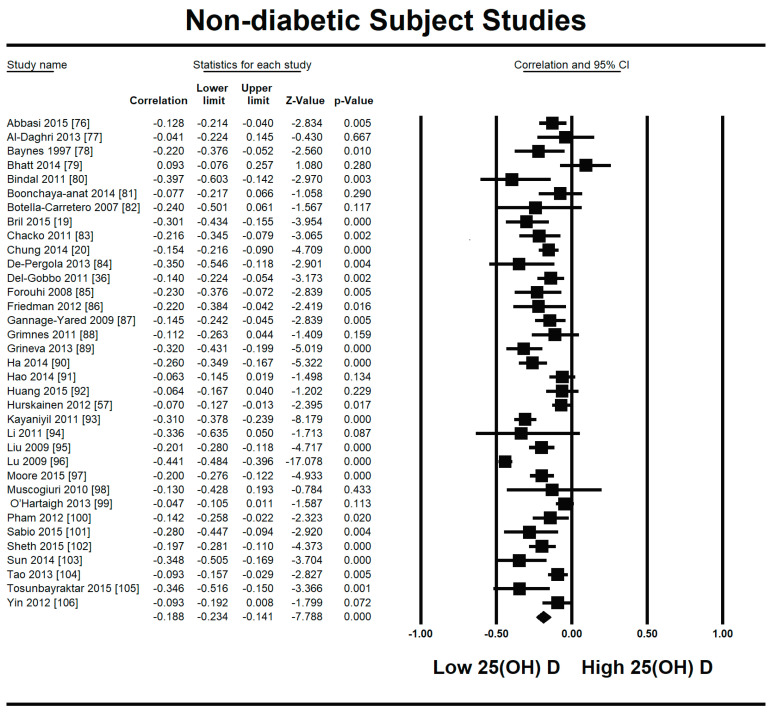
Forest plot for non-diabetic subject studies showing the correlation between status of vitamin D and fasting plasma insulin. The random effect model was used for the determination of correlation and 95% CI [19,20,57,76,77,78,79,80,81,82,83,84,85,86,87,88,89,90,91,92,93,94,95,96,97,98,99,100,101,102,103,104,105,106].

**Figure 3:**
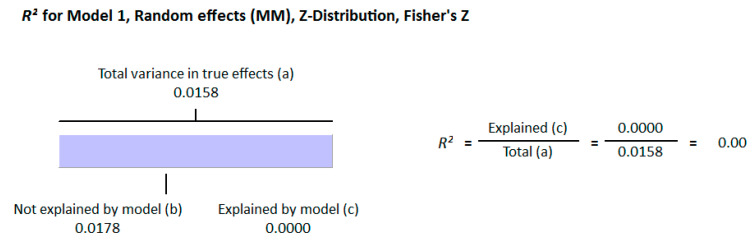
Meta-regression analysis for non-diabetic subject studies. Latitude R^2^ shows the effect of the moderator on the heterogeneity of correlation. (**a**) total variance in true effects; (**b**) not explained by model; (**c**) explained by model.

**Figure 4:**
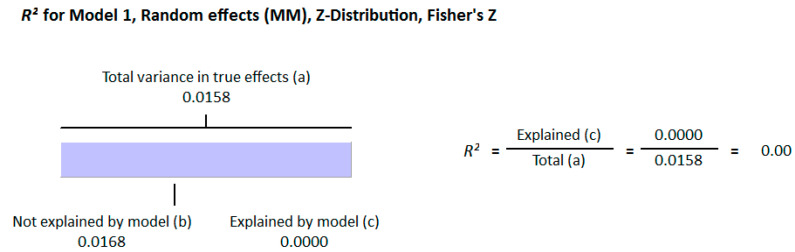
Meta-regression analysis for non-diabetic subject studies. Method of determination of vitamin D R^2^ shows the effect of the moderator on the heterogeneity of correlation. (**a**) total variance in true effects; (**b**) not explained by model; (**c**) explained by model.

**Figure 5:**
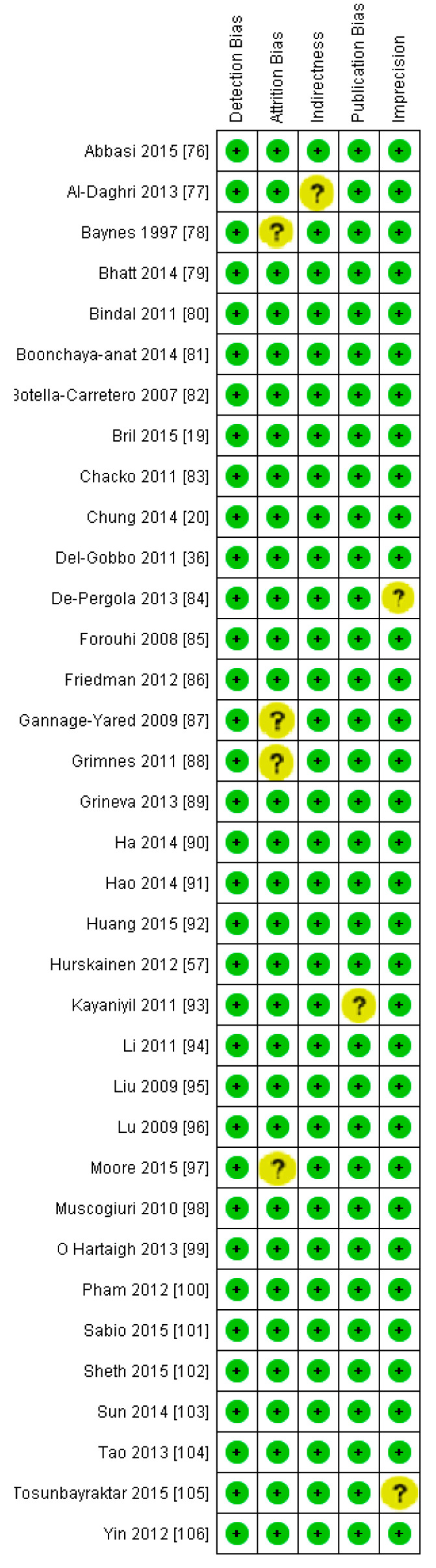
Risk of bias assessment for non-diabetic subject studies (plus sign shows low risk, minus sign shows high risk, and question mark shows unknown bias) [19,20,36,57,76,77,78,79,80,81,82,83,84,85,86,87,88,89,90,91,92,93,94,95,96,97,98,99,100,101,102,103,104,105,106].

**Figure 6:**
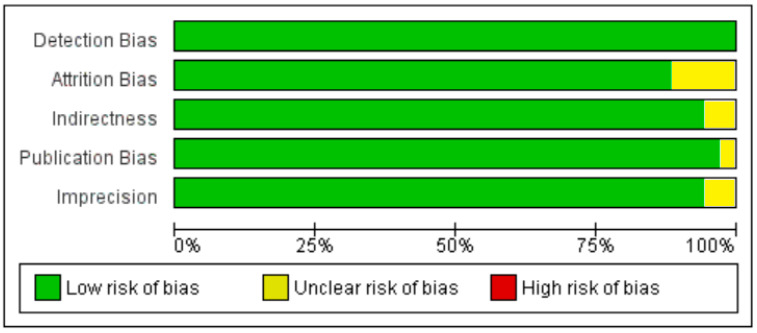
Summary of risk of bias assessment for non-diabetic subject studies, data shown are in percentages.

**Figure 7:**
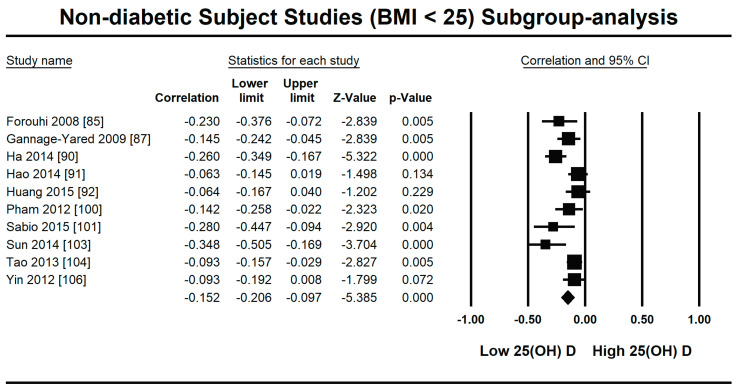
Forest plot for the lowest BMI quartile (<25) of non-diabetic subject studies showing the correlation between the status of vitamin D and fasting plasma insulin. The random effects model was used for the determination of correlation and 95% CI [85,87,90,91,92,100,101,103,104,106].

**Figure 8:**
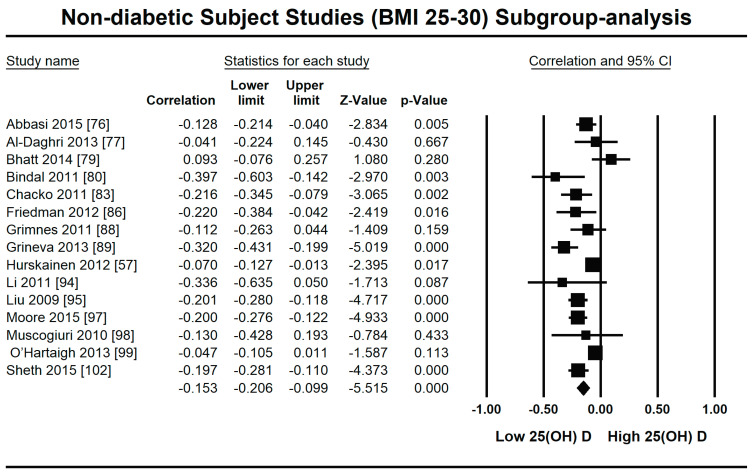
Forest plot for the medium BMI quartile (25–30) of non-diabetic subject studies showing the correlation between the status of vitamin D and fasting plasma insulin. The random effects model was used for the determination of correlation and 95% CI [57,76,77,79,80,83,86,88,89,94,95,97,98,99,102].

**Figure 9:**
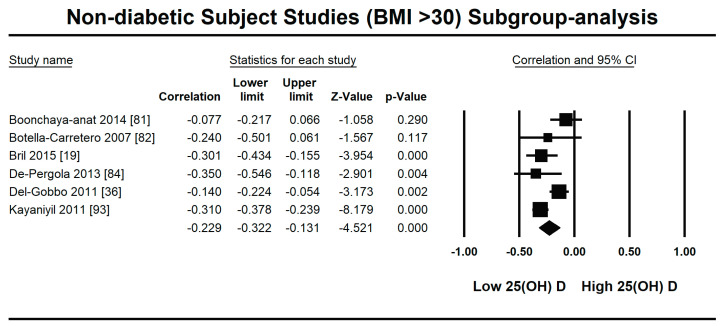
Forest plot for the highest BMI quartile (>30) of non-diabetic subject studies showing the correlation between the status of vitamin D and fasting plasma insulin. The random effects model was used for the determination of correlation and 95% CI [19,36,81,82,84,93].

Because of the large amount of variability due to the above-mentioned sources, we used the random effect model for this meta-analysis. An inverse relationship (*r* = −0.188, 95% CI = −0.141 to −0.234, *p* = 0.000) was seen between fasting plasma insulin and vitamin D concentrations in the blood for all thirty-five non-diabetic subject studies (Figure 2). The correlation of all studies lies between *r* = −0.041 and *r* = −0.397. The meta-regression analysis showed R^2^ to be zero for both latitude and method of determination of vitamin D, meaning the relationship between vitamin D concentration and fasting plasma insulin is independent of these two variables (Figure 3 and Figure 4). The summary of the GRADE assessments is presented in Figure 5 and Figure 6. The subgroup analysis for different quartiles of BMI depicts an overall increasing strength of correlation between fasting plasma insulin and vitamin D status from lower to higher BMI quartile. For example, the correlation was *r* = −0.152, 95% = −0.206 to −0.097, *p* = 0.000 in the lowest quartile (BMI < 25) (Figure 7); *r* = −0.153, 95% = −0.206 to −0.099, *p* = 0.000 in the medium quartile; and *r* = −0.229, 95% = −0.322 to −0.131 (BMI = 25–30) (Figure 8), *p* = 0.000 in the highest quartile (BMI > 25) (Figure 9). The correlation was almost the same in the first two quartiles; however, it was significantly higher in the third quartile compared to the first two quartiles.

#### 3.2.2. Meta-Analysis and Meta-Regression for Diabetes Patient Studies

**Figure 10:**
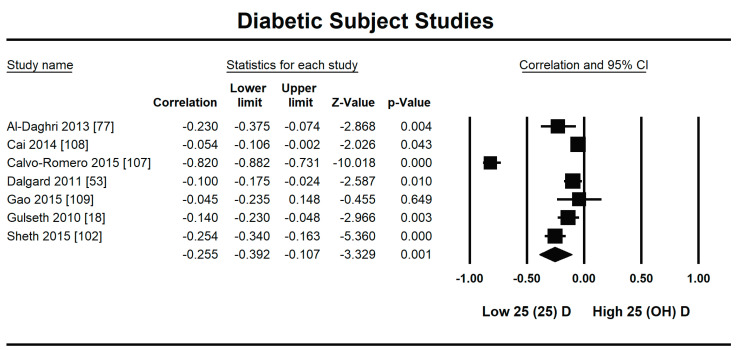
Forest plot for diabetic subject studies showing the correlation between status of vitamin D and fasting plasma insulin. The random effects model was used for the determination of correlation and 95% CI [18,53,77,107,108,109].

**Figure 11:**
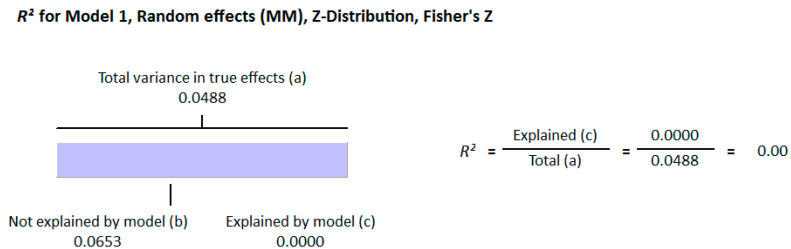
Meta regression analysis for diabetic subject studies. Latitude R^2^ shows the effect of the moderator on the heterogeneity of correlation. (**a**) total variance in true effects; (**b**) not explained by model; (**c**) explained by model.

**Figure 12:**
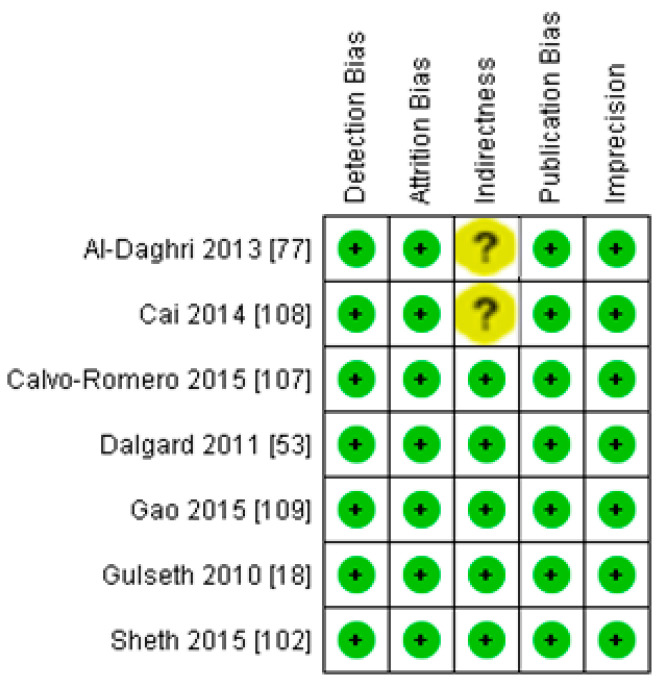
Risk of bias assessment for diabetic subject studies (plus sign shows low risk, minus sign shows high risk, and question mark shows unknown bias) [18,53,77,102,107,108,109].

**Figure 13:**
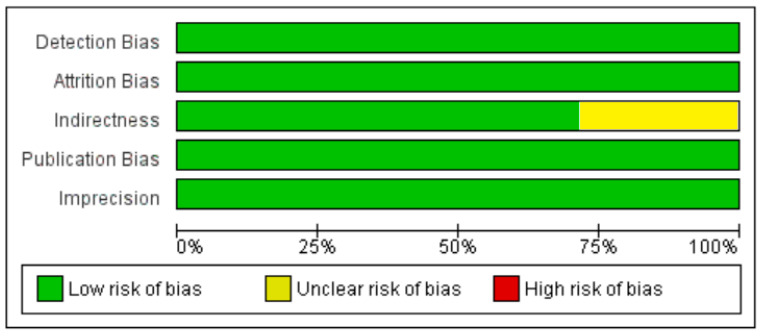
Summary of risk of bias assessment for diabetic subject studies: data shown are in percentages.

Seven studies fulfilled the criteria to be included in this meta-analysis for the relationship of vitamin D with fasting plasma insulin in diabetic patients. In this meta-analysis, we found an inverse association (*r* = −0.255, 95% CI = −0.392 to −0.107, *p* = 0.001) between fasting plasma insulin and vitamin D levels (Figure 10). The range of correlation in all studies was −0.045 to −0.25, except for one study from Southern Spain [107], which showed an increased correlation (*r* = −0.882). The effect of the moderator (latitude) on the correlation of vitamin D status and fasting plasma insulin was determined by meta-regression analysis. The results showed that the latitude (R^2^ = 0.000%, *p* = 0.000) did not contribute to heterogeneity in this correlation (Figure 11). The summary of GRADE assessment is presented in Figure 12 and Figure 13.

## 4. Discussion

It is evident from this meta-analysis that the levels of vitamin D in the body are inversely related to insulin resistance both in diabetic and non-diabetic populations. However, the correlation is stronger in the diabetic population (*r* = −0.255, 95% CI = −0.392 to −0.107, *p* = 0.001) (Figure 2) compared with the non-diabetic population (*r* = −0.188, 95% CI = −0.141 to −0.234, *p* = 0.000) (Figure 10).

The status of vitamin D is inversely related to insulin resistance independent of age and sex. The active form of vitamin D (1,25-hydroxy vitamin D) has been detected in the pancreas [110]; therefore, there is a possibility that vitamin D plays a role in the evolutionary development of metabolic systems such as beta cell function. Hypovitaminosis D is associated with reduced calcium status in the blood circulation, which ultimately controls insulin synthesis and insulin secretion by beta cells [111]. Vitamin D supplementation increases plasma calcium levels, which in turn increase the synthesis and secretion of calcium from the beta cells, ultimately improving glucose homeostasis [21,73]. Hypovitaminosis D therefore plays a role in the development of insulin resistance by affecting insulin synthesis and secretion from beta cells and by regulating circulating serum calcium.

The subgroup analysis on the basis of BMI showed an increasingly strong inverse relationship between vitamin D status and insulin resistance with increasing BMI in non-diabetic subject studies. The strength of correlation is stronger (*r* = −0.229, 95% = −0.322 to −0.131) in the highest BMI quartile, and almost the same in the first (*r* = −0.152, 95% = −0.206 to −0.097, *p* = 0.000) and second (*r* = −0.153, 95% = −0.206 to −0.099, *p* = 0.000) BMI quartiles. According to previous studies, a synergy exists between hypovitaminosis D and obesity in developing insulin resistance [14,20,42]. The expression of vitamin D receptors is more pronounced in obese compared with lean subjects, and vitamin D deficiency has an independent inverse relationship with BMI [112]. The anti-insulin resistance mechanism of vitamin D might act through its anti-inflammatory mechanism in overweight subjects. A decrease in inflammatory cytokines after vitamin D treatment has been observed in many previous studies and might have a role in promoting insulin sensitivity [21]. The cycle works via insulin-stimulated fat synthesis and adipose tissue initiating the synthesis of inflammatory markers, which then lead to augmented insulin resistance. Vitamin D interrupts this cycle at the level of adipogenesis by hindering it and at the level of inflammatory marker production by lowering their synthesis [113].

The underlying cause of obesity-related insulin resistance is inflammation induced by obesity. Vitamin D is well-known for its anti-inflammatory functions as it lowers the concentration of different inflammatory indicators (C-reactive protein (CRP), tumor necrosis factor-a (TNF-alpha), and interleukin-6 (IL-6)) [114]. Numerous studies have shown the effect of insulin resistance on the risk of cardiovascular disease, which is doubled in insulin-resistant compared with normal populations. Considerable similarities in the biochemical profile of insulin resistance and inflammation have been observed in diabetic and cardiovascular patients recently. A recent study even showed a role for insulin resistance in the development of ischemic heart disease under normal glucose tolerance [115].

Vitamin D receptor (VDR) is required for the functioning of vitamin D in different tissues. However, the requirements for the expression of VDR vary in different tissues, e.g., in some tissues, it requires calcium and vitamin D for its expression, and in others, it needs neither. It has been reported that vitamin D induces insulin secretion in the beta cells of the pancreas and increases insulin sensitivity in target cells, i.e., muscle, adipose tissue, and liver [116,117,118]. Hypovitaminosis D has been shown to be related to hyperglycemia and insulin resistance earlier [29,119].

The epigenetic effect of vitamin D has been observed at the level of transcription for many genes. Insulin receptor substrate (IRS-1) is a protein that plays an important role in promoting insulin sensitivity. The expression of IRS protein was observed to be increased by 2.4 times in high-fat-treated mouse muscle tissue after treatment with vitamin D. The anti-insulin resistance mechanism of vitamin D appears to involve insulin-mediated intracellular functions through IRS-1 [120]. The photosynthetic production of vitamin D in the skin depends on the radiation (UV-B) from sunlight. Therefore, latitude can explain the status of vitamin D geographically, but the meta-regression analysis presented in this study does not show any variability in the correlation because of latitude (R^2^ = 0.000, *p* = 0.000). This might be because many factors in the modern world have reduced the impact of these radiations on the production of vitamin D. For example, concrete buildings absorb more radiation, and the gases emitted by industry and vehicles reduce the irradiance of ultraviolet B radiation from the sun [121,122]. These and other factors, such as diet, clothing styles, industrialization, reduced time for sun exposure, and skin pigmentation, have confounded the effect of latitude on the strength of correlation between vitamin D status and insulin resistance.

The meta-regression analysis for the effect of method of determination of vitamin D also showed no heterogeneity of the correlation. However, we observed an overall increased strength of inverse correlation between vitamin D status and insulin resistance when the CLIA method was used for the determination of vitamin D; the most pronounced example being the study of Calvo-Romero [107] from Southern Spain, which reported the highest correlation of −0.82.

Vitamin D is directly related to the progression of the diabetic complications such as diabetic neuropathy, diabetic nephropathy, and diabetic retinopathy. Vitamin D regulates neurotrophin and calcium homeostasis related to nerve action, and the deficiency of vitamin D exerts diverse effects on the complication of diabetic neuropathy [123]. It was observed previously that vitamin D is inversely related to diabetic neuropathy, and this relationship does not depend on the duration of diabetes disease. Chronic nephropathy developed during type 2 diabetes was also linked to diabetic neuropathy [124]. The role of vitamin D in the functioning of neurons has been established in the last couple of decades. Many neuronal diseases have been proven to be associated with hypovitaminosis D. For example, treating multiple sclerosis patients with vitamin D can slow the progression of disability [125,126,127]. Nerve growth factor is important for the growth and development of neurons, and myelination of Schwann cells in case of injury. Vitamin D increases the production of nerve growth factor in glial cells after crossing the blood–brain barrier and entering the glial cells [128,129,130]. There was progress in the treatment of diabetic foot healing when the diabetic foot was topically treated with nerve growth factor [131].

### Strengths and Weaknesses

The systematic search used for the mining of research articles is one of the major strengths of this meta-analysis. The gold standard international methodology was applied, and observational studies were evaluated by the Grading of Recommendations Assessment, Development, and Evaluation (GRADE). The meta-analysis did not reveal very wide 95% confidence intervals, which shows the dependence of insulin resistance on the status of vitamin D. Although the total number of subjects was high in this meta-analysis, the studies were observational; therefore, the chances for residual confounding cannot be ruled out, which is a limitation. Potentially confounding factors include the age, ethnicity, and lifestyle of the participants. The intake of vitamin D and sun exposure has not been mentioned in all of the studies, which may be an additional source of confounding. Observational studies have the drawback of not being blinded and randomized, which is a limitation of this study. We consider this evidence to be moderate on the basis of the strengths and weaknesses of the studies included.

## 5. Conclusions

Diabetic hypovitaminosis D is at the pandemic level worldwide. The present systematic review and meta-analysis suggest a role of vitamin D in the regulation of insulin production and release from the beta cells of Langerhans. However, this association is not purely independent, and strongly depends on BMI as observed in the subgroup-analysis. The inverse correlation between vitamin D status and fasting insulin strengthens with increasing BMI. The meta-regression analysis did not show any effect of latitude or the method of determination of vitamin D on the overall relationship of vitamin D levels in the body and fasting insulin in the blood. There is a significant need for high-quality, long-term, randomized controlled trials to be conducted using different doses of vitamin D to see its effect on fasting plasma insulin levels.

## Data Availability

Not applicable.

## References

[1] Kahn S.E., Hull R.L., Utzschneider K.M. (2006). Mechanisms Linking Obesity to Insulin Resistance and Type 2 Diabetes. Nature.

[2] Ravier M.A., Rutter G.A. (2006). Glucose or Insulin, but not Zinc Ions, Inhibit Glucagon Secretion from Mouse Pancreatic Alpha-cells. Diabetes.

[3] Williamson J.R., Browning E.T., Olson M. (1968). Interrelations between Fatty Acid Oxidation and the Control of Gluconeogenesis in Perfused Rat Liver. Adv. Enzym. Regul..

[4] Dunaif A. (1997). Insulin Resistance and the Polycystic Ovary Syndrome: Mechanism and Implications for Pathogenesis. Endocr. Rev..

[5] Marchesini G., Brizi M., Morselli-Labate A.M., Bianchi G., Bugianesi E., McCullough A.J., Forlani G., Melchionda N. (1999). Association of Nonalcoholic Fatty Liver Disease with Insulin Resistance. Am. J. Med..

[6] Petersen K.F., Oral E.A., Dufour S., Befroy D., Ariyan C., Yu C., Cline G.W., DePaoli A.M., Taylor S.I., Gorden P. (2002). Leptin Reverses Insulin Resistance and Hepatic Steatosis in Patients with Severe Lipodystrophy. J. Clin. Investig..

[7] Kahn S.E. (2003). The relative contributions of insulin resistance and beta-cell dysfunction to the pathophysiology of Type 2 diabetes. Diabetologia.

[8] Kasuga M. (2006). Insulin Resistance and Pancreatic Beta Cell Failure. J. Clin. Investig..

[9] Schwartz S.S., Epstein S., Corkey B.E., Grant S.F.A., Gavin I.J.R., Aguilar R.B., Herman M.E. (2017). A Unified Pathophysiological Construct of Diabetes and its Complications. Trends Endocrinol. Metab..

[10] Al Mheid I., Patel R.S., Tangpricha V., Quyyumi A.A. (2013). Vitamin D and Cardiovascular Disease: Is the Evidence Solid?. Eur. Heart J..

[11] Tomson J., Emberson J., Hill M., Gordon A., Armitage J., Shipley M. (2013). Vitamin D and Risk of Death from Vascular and Non-vascular Causes in the Whitehall Study and Meta-analyses of 12,000 Deaths. Eur. Heart J..

[12] Seo J.A., Eun C.R., Cho H., Lee S.K., Yoo H.J., Kim S.G. (2013). Low Vitamin D Status is Associated with Non-alcoholic Fatty Liver Disease Independent of Visceral Obesity in Korean Adults. PLoS ONE.

[13] Dattola A., Silvestri M., Bennardo L., Passante M., Scali E., Patruno C., Nistico S.P. (2020). Role of Vitamins in Skin Health: A Systematic Review. Curr. Nutr. Rep..

[14] Chiu K.C., Chu A., Go V.L.W., Saad M.F. (2004). Hypovitaminosis D is Associated with Insulin Resistance and Cell Dysfunction. Am. J. Clin. Nutr..

[15] Pittas A.G., Lau J., Hu F.B. (2007). Dawson-Hughes, B. The Role of Vitamin D and Calcium in Type 2 Diabetes. A Systematic Review and Meta-Analysis. J. Clin. Endocrinol. Metab..

[16] Kositsawat J., Freeman V., Gebber B., Geraci S. (2010). Association of A1c Levels with Vitamin D Status in U.S. Adults. Diabetes Care.

[17] Hypponen E., Power C. (2006). Vitamin D Status and Glucose Homeostasis in the 1958 British Bird Cohort: The role of obesity. Diabetes Care.

[18] Gulseth H.L., Gjelstad I.M.F., Tierney A.C., Lovengrove J.A., Defoort C., Blaak E.E., Lopez-Miranda J., Kiec-Wilk B., Ris U., Roshe H. (2010). Serum Vitamin D Concentration Does Not Predict Insulin Action or Secretion in European Subjects with the Metabolic Syndrome. Diabetes Care.

[19] Bril F., Maximos M., Portillo-Sanchez P., Biernacki D., Lomonaco R., Subbarayan S., Correa M., Lo M., Suman A., Cusi K. (2015). Relationship of Vitamin D with Insulin Resistance and Disease Severity in Non-alcoholic Steatohepatitis. J. Hepatol..

[20] Chung S.J., Lee Y.A., Hong H., Kang M.J., Kwon H.J., Shin C.H., Yang S.W. (2014). Inverse Relationship between Vitamin D Status and Insulin Resistance and the Risk of Impaired Fasting Glucose in Korean Children and Adolescents: The Korean National Health and Nutrition Examination Survey (KNHANES) 2009–2010. Public Health Nutr..

[21] Alvarez J.A., Ashraf A. (2010). Role of Vitamin D in Insulin Secretion and Insulin Sensitivity for Glucose Homeostasis. Int. J. Endocrinol..

[22] Dutta D., Maisnam I., Shrivastava A., Sinha A., Ghosh S., Mukhopadhyay P., Mukhopadhyay S., Chowdhury S. (2013). Serum Vitamin-D Predicts Insulin Resistance in Individuals with Prediabetes. Indian J. Med. Res..

[23] Kabadi S.M., Lee B.K., Liu L. (2012). Joint Effects of Obesity and Vitamin D Insufficiency on Insulin Resistance and Type 2 Diabetes: Results from the NHANES 2001–2006. Diabetes Care.

[24] Kim S., Lim J., Kye S., Joung H. (2012). Association between Vitamin D Status and Metabolic Syndrome Risk among Korean Population: Based on the Korean National Health and Nutrition Examination Survey IV-2, 2008. Diabetes Res. Clin. Pract..

[25] Kobzaa V.M., Feet J.C., Zhoua J., Conley T.B., Peacock M., Reger H.B.I., Palmad G.D., Campbell W.W. (2013). Vitamin D Status and Resistance Exercise Trainingindependently Affect Glucose Tolerance in Older Adults. Nutr. Res..

[26] Liu J., Tan J., Jeynes B. (2013). Serum 25(OH) Vitamin D Level, Femur Length, and Risk of Type 2 Diabetes among Adults. Appl. Physiol. Nutr. Metab..

[27] Nguyen V.T., Li X., Elli E.F., Ayloo S.M., Castellanos K.J., Fantuzzi G., Freels S., Braunschweig C.L. (2015). Vitamin D, Inflammation, and Relations to Insulin Resistance in Premenopausal Women with Morbid Obesity. Obesity.

[28] Pannu P.K., Piers L.S., Soares M.J., Zhao Y., Ansari Z. (2017). Vitamin D Status is Inversely Associated with Markers of Risk for Type 2 Diabetes: A Population Based Study in Victoria, Australia. PLoS ONE.

[29] Scragg R., Sowers M.R., Bell C. (2004). Serum 25-Hydroxyvitamin D, Diabetes, and Ethnicity in the Third National Health and Nutrition Examination Survey. Diabetes Care.

[30] Weiler H.A., Lowea J., Krahnb J., William D. (2013). LesliecOsteocalcin and Vitamin D Status are Inversely Associated with Homeostatic Model Assessment of Insulin Resistance in Canadian Aboriginal and White Women: The First Nations Bone Health Study. J. Nutr. Biochem..

[31] Alkharfy K.M., Al-Daghri N.M., Sabico S.B., Al-Othman A., Moharram O., Alokail M.S., Al-Saleh Y., Kumar S., Chrousos G.P. (2013). Vitamin D Supplementation in Patients with Diabetes Mellitus Type 2 on Different Therapeutic Regimens: A One-year Prospective Study. Cardiovasc. Diabetol..

[32] Al-Shoumer K.A., Al-Asoosi A.A., Ali A.H., Nair V.S. (2013). Does Insulin Resistance in Type 2 Diabetes Alter Vitamin D Status?. Prim. Care Diabetes.

[33] Gedik O., Akalin S. (1986). Effects of Vitamin D Deficiency and Repletion on Insulin and Glucagon Secretion in Man. Diabetologia.

[34] Bardini G., Giannini S., Romano D., Rotella C.M., Mannucci E. (2013). Lipid Accumulation Product and 25-OH-Vitamin D Deficiency in Type 2 Diabetes. Rev. Diabet. Stud..

[35] Nimitphong H., Chailurkit L., Chanprasertyothin S., Sritara P., Ongphiphadhanakul B. (2013). The Association of Vitamin D Status and Fasting Glucose According to Body Fat Mass in Young Healthy Thais. Endocr. Disord..

[36] Del-Gobbo L.C., Song Y., Dannenbaum D.A., Dewailly E., Egeland G.M. (2011). Serum 25-Hydroxyvitamin D Is not Associated with Insulin Resistance or Beta Cell Function in Canadian Cree. J. Nutr..

[37] Diaz G.M., Gonza L., Ramos-Trautmann G., Marie C., Palacios C. (2016). Vitamin D Status Is Associated with Metabolic Syndrome in a Clinic-Based Sample of Hispanic Adults. Metab. Syndr. Relat. Disord..

[38] Hidayat R., Setiati S., Soewondo P. (2010). The Association Between Vitamin D Deficiency and Type 2 Diabetes Mellitus in Elderly Patients. Age.

[39] Hirani V., Cumming R.G., Le Couteur D.G., Naganathan V., Blyth F., Handelsman D.J., Waite L.M., Seibel M.J. (2014). Low Levels of 25-Hydroxy Vitamin D and Active 1,25-Dihydroxyvitamin D Independently Associated with Type 2 Diabetes Mellitus in Older Australian Men: The Concord Health and Ageing in Men Project. J. Am. Geriatr. Soc..

[40] Husemoen L.L., Thuesen B.H., Fenger M., Jorgensen T., Glumer C., Svensson J., Ovesen L., Witte D.R., Linneberg A. (2012). Serum 25(OH)D and Type 2 Diabetes Association in a General Population: A Prospective Study. Diabetes Care.

[41] Justice J.N., Pierpoint L.A., Mani D., Schwartz R.S., Enoka R.M. (2014). Motor Function is Associated with 1,25(OH)2D and Indices of Insulin–glucose Dynamics in Non-diabetic Older Adults. Aging Clin. Exp. Res..

[42] Kabadi S.M., Liu L., Auchincloss A.H., Zakeri I.F. (2013). Multivariate Path Analysis of Serum 25-hydroxyvitamin D Concentration, Inflammation, and Risk of Type 2 Diabetes Mellitus. Dis. Mark..

[43] Khor G.L., Chee W.S.S., Shariff Z.M., Poh B.K., Arumugam M., Rahman J.A., Theobaldb H.E. (2011). High Prevalence of Vitamin D Insufficiency and Its Association with BMI-for-age among Primary School Children in Kuala Lumpur, Malaysia. BMC Public Health.

[44] Lee B., Park S., Kim Y. (2012). Age- and Gender-specific Associations between Low Serum 25-hydroxyvitamin D Level and Type 2 Diabetes in the Korean General Population: Analysis of 2008–2009 Korean National Health and Nutrition Examination Survey data. Asia Pac. J. Clin. Nutr..

[45] Li L., Yin X., Yao C., Zhu X., Wu X. (2013). Serum 25-hydroxyvitamin D, Parathyroid Hormone, and Their Association with Metabolic Syndrome in Chinese. Endocrine.

[46] Lu L., Wu Y., Qi Q., Liu C., Gan W. (2012). Associations of Type 2 Diabetes with Common Variants in PPARD and the Modifying Effect of Vitamin D among Middle-Aged and Elderly Chinese. PLoS ONE.

[47] Marques-Vidal P., Vollenweider P., Guessous I., Henry H., Boulat O., Waeber G., Jornayvaz F.R. (2015). Serum Vitamin D Concentrations Are Not Associated with Insulin Resistance in Swiss Adults. J. Nutr..

[48] Tsur A., Feldman B.S., Feldhammer I., Hoshen M.B., Leibowitz G., Balicer R.D. (2013). Decreased Serum Concentrations of 25-hydroxycholecalciferol are Associated with Increased Risk of Progression to Impaired Fasting Glucose and Diabetes. Diabetes Care.

[49] Wright O.R.L., Hickman I.J., Petchey W.G., Sullivan C.M., Ong C., Rose F.J., Ng C., Prins J.B., Whitehead J.P., Moore-Sullivan T.M. (2013). The Effect of 25-hydroxyvitamin D on Insulin Sensitivity in Obesity: Is It Mediated via Adiponectin?. Can. J. Physiol. Pharmacol..

[50] Wright C.S., Weinheimer-Haus E.M., Fleet J.C., Peacock M., Campbell W.W. (2015). The Apparent Relation between Plasma 25-Hydroxyvitamin D and Insulin Resistance Is Largely Attributable to Central Adiposity in Overweight and Obese Adults. J. Nutr..

[51] Zoppini G., Galletti A., Targher G., Brangani C., Pichiri I., Negri C., Stoico V., Cacciatori V., Bonora E. (2013). Glycated Haemoglobin Is Inversely Related to Serum Vitamin D Levels in Type 2 Diabetic Patients. PLoS ONE.

[52] Bellan M., Guzzaloni G., Rinaldi M., Merlotti E., Ferrari C., Tagliaferri A., Pirisi M., Aimaretti G., Scacchi M., Marzullo P. (2014). Altered Glucose Metabolism Rather Than Naïve Type 2 Diabetes Mellitus (T2DM) is Related to Vitamin D Status in Severe Obesity. Cardiovasc. Diabetol..

[53] Dalgard C., Skaalum M., Weihe P.P., Grandjean P. (2011). Vitamin D Status in Relation to Glucose Metabolism and Type 2 Diabetes in Septuagenarians. Diabetes Care.

[54] Heras J.D.L., Rajkumar K., Lee S., Bacha F., Holick M.F., Arslanian S.A. (2013). 25-Hydroxyvitamin D in Obese Youth Across theSpectrumofGlucose Tolerance from Normal to Prediabetes to Type 2 Diabetes. Diabetes Care.

[55] Esteghamatia A., Aryanab B., Esteghamatia A., Nakhjavania M. (2014). Differences in Vitamin D Concentration between Metabolically Healthy Andunhealthy Obese Adults: Associations with Inflammatory and Cardiometabolicmarkers in 4391 Subjects. Diabetes Metab..

[56] Huang Y., Li X., Wang M., Ning H., Lima A., Li Y., Sun C. (2013). Lipoprotein lipase links vitamin D, insulin resistance, and type 2 diabetes: A cross-sectional epidemiological study. Cardiovasc. Diabetol..

[57] Hurskainen A.R., Virtanen J.K., Tuomainen T.P., Nurmi T., Voutilainen S. (2012). Association of Serum 25-hydroxyvitamin D with Type 2 Diabetes and Markers of Insulin Resistance in a General Older Population in Finland. Diabetes Metab. Res. Rev..

[58] Jiang H., Peng S. (2014). The Relationship between Serum Vitamin D and HOMA-IR in Overweight Elderly Patients. Int. J. Cardiol..

[59] Park H.Y., Lim Y., Kim J.H., Bae S., Oh S., Hong Y. (2012). Association of Serum 25-Hydroxyvitamin D Levels with Markers for Metabolic Syndrome in the Elderly: A Repeated Measure Analysis. J. Korean Med. Sci..

[60] Kavadar G., Demircioglu D.T., Ozgonenel L., Emre T.Y. (2015). The Relationship between Vitamin D Status, Physical Activity and Insulin Resistance in Overweight and Obese Subjects. Bosn. J. Basic Med. Sci..

[61] Kim J. (2015). Association between Serum Vitamin D, Parathyroid Hormone and Metabolic Syndrome in Middle-aged and Older Korean Adults. Eur. J. Clin. Nutr..

[62] Kim M.K., Kang M.I., Oh K.W., Kwon H.S., Lee J.H., Lee W.C., Yoon K., Ho Y. (2010). The Association of Serum Vitamin D Level with Presence of Metabolic Syndrome and Hypertension in Middle-aged Korean Subjects. Clin. Endocrinol..

[63] Lim S., Kim M.J., Choi S.H., Shin C.S., Park K.S., Jang H.C., Billings L.K., Meigs J.B. (2013). Association of Vitamin D Deficiency with Incidence of Type 2 Diabetes in High-risk Asian Subjects. Am. J. Clin. Nutr..

[64] Morisset A., Tardio V., Weisnagel J., Lemieux S., Bergeron J., Gagnon C. (2015). Associations Between Serum 25-Hydroxyvitamin D, Insulin Sensitivity, Insulin Secretion, and B-Cell Function According to Glucose Tolerance Status. Metab. Syndr. Relat. Disord..

[65] Nielsen N.O., Bjerregaard P., Rønn P.F., Friis H., Andersen S., Melbye M. (2016). Associations between Vitamin D Status and Type 2 Diabetes Measures among Inuit in Greenland May Be Affected by Other Factors. PLoS ONE.

[66] Pinelli N.R., Jaber L.A., Brown M.B., Herman W.H. (2010). 3Serum 25-Hydroxy Vitamin D and Insulin Resistance, Metabolic Syndrome, and Glucose Intolerance Among Arab Americans. Diabetes Care.

[67] Rhee S.Y., Hwang Y.C., Chung H.Y., Woo J.T. (2012). Vitamin D and Diabetes in Koreans: Analyses Based on the Fourth Korea National Health and Nutrition Examination Survey (KNHANES), 2008–2009. Diabetic Med..

[68] Esteghamati A., Aryan Z., Esteghamati A.R., Nakhjavani M. (2015). Vitamin D Deficiency is Associated with Insulin Resistance in Nondiabetics and Reduced Insulin Production in Type 2 Diabetics. Horm. Metab. Res..

[69] Al-Daghri N.M., Al-Attas O.S., Al-Okail M.S., Alkharfy K.M., Al-Yousef M.A., Nadhrah H.M., Sabico S.B., Chrousos G.P. (2010). Severe Hypovitaminosis D is Widespread and more Common in Non-diabetics Than Diabetics in Saudi Adults. Saudi Med. J..

[70] Eraslan S., Kizilgul M., Uzunlulu M., Colak Y., Ozturk O., Tuncer I. (2013). Frequency of Metabolic Syndrome and 25-hydroxyvitamin D3 Levels in Patients with Non-alcoholic Fatty Liver Disease. Minerva Med..

[71] Hutchinson M.S., Figenschau Y., Almås B., Njølstad I., Jorde R. (2011). Serum 25-hydroxyvitamin D Levels in Subjects with Reduced Glucose Tolerance and Type 2 Diabetes–The Tromsø OGTT-study. Int. J Vitam. Nutr. Res..

[72] Imura H., Seino Y., Ishida H. (1985). Osteopenia and Circulating Levels of Vitamin D Metabolites in Diabetes Mellitus. J. Nutr. Sci. Vitaminol..

[73] Nomata S., Kadowaki S., Yamatani T., Fukase M., Fujita T. (1986). Effect of 1 Alpha (OH)-vitamin D3 on Insulin Secretion in Diabetes Mellitus. Bone Miner..

[74] Mhatre M., Hall M. (2010). Student Forum: Does Calcium and Vitamin D Intake Affect Incidence of Type 2 Diabetes Mellitus and Insulin Resistance Syndrome?. Consult. Pharm..

[75] Mirzaei K., Hossein-Nezhad A., Keshavarz S.A., Eshaghi S.M., Koohdani F., Saboor-Yaraghi A.A., Hosseini S., Tootee A., Djalali M. (2014). Insulin Resistance via Modification of PGC1α Function Identifying a Possible Preventive Role of Vitamin D Analogues in Chronic Inflammatory State of Obesity. A Double Blind Clinical Trial Study. Minerva Med..

[76] Abbasi F., Blasey C., Feldman D., Caulfield M.P., Hantash F.M., Reaven G.M. (2015). Low Circulating 25-Hydroxyvitamin D Concentrations Are Associated with Defects in Insulin Action and Insulin Secretion in Persons with Prediabetes. J. Nutr..

[77] Al-Daghri N.M., Al-Attas O.S., Alokail M.S., Alkharfy K.M., Al-Othman A., Draz H.M., Yakout S.M., Al-Saleh Y., Al-Yousef M., Sabico S. (2013). Hypovitaminosis D Associations with Adverse Metabolic Parameters are Accentuated in Patients with Type 2 Diabetes Mellitus: A Body Mass Index-independent Role of Adiponectin?. J. Endocrinol. Investig..

[78] Baynes K.C.R., Boucher B.J., Feskens E.J.M., Kromhout D. (1997). Vitamin D Glucose Tolerance and Insulinaemia in Elderly Men. Diabetologia.

[79] Bhatt S.P., Misra A., Sharma M., Guleria R., Pandey R.M., Luthra K., Vikram N.K. (2014). Vitamin D Insufficiency is Associated with Abdominal Obesity in Urban Asian Indians without Diabetes in North India. Diabetes Technol. Ther..

[80] Bindal M.E., Taskapan H. (2011). Hypovitaminosis D and Insulin Resistance in Peritoneal Dialysis Patients. Int. Urol. Nephrol..

[81] Boonchaya-anant P., Holick F.M., Caroline M. (2014). ApovianSerum 25-Hydroxyvitamin D Levels and Metabolic Health Status in Extremely Obese Individuals. Obesity.

[82] Botella-Carretero J.I., Alvarez-Blasco F., Villafruela J.J., Balsa J.A., Vazquez C., Escobar-Morreale H.F. (2007). Vitamin D Deficiency is Associated with the Metabolic Syndrome in Morbid Obesity. Clin. Nutr..

[83] Chacko S.A., Song Y., Manson J.E., Horn L.V., Eaton C., Martin L.W., McTiernan A., Curb J.D., Wylie-Rosett J., Phillips L.S. (2011). Serum 25-hydroxyvitamin D Concentrations in Relation to Cardiometabolic Risk Factors and Metabolic Syndrome in Postmenopausal Women. Am. J. Clin. Nutr..

[84] De-Pergola G., Nitti A., Bartolomeo N., Gesuita A., Giagulli V.A., Triggiani V., Guastamacchia E., Silvestris F. (2013). Possible Role of Hyperinsulinemia and Insulin Resistance in Lower Vitamin D Levels in Overweight and Obese Patients. BioMed Res. Int..

[85] Forouhi N.G., Luan J., Cooper A., Boucher B.J., Wareham N.J. (2008). Baseline Serum 25-hydroxy Vitamin D is Predictive of Future Glycemic Status and Insulin Resistance. Diabetes.

[86] Friedman D.J., Bhatt N., Hayman N.S., Nichols B.J., Herman M., Nikolaev N., Danziger J. (2012). Impact of Activated Vitamin D on Insulin Resistance in Nondiabetic Chronic Kidney Disease Patients. Clin. Endocrinol..

[87] Gannage-Yared M.L., Chedid R., Khalife S., Azzi E., Zoghbi F., Halaby G. (2009). Vitamin D in Relation to Etabolic Risk Factors, Insulin Sensitivity and Adiponectin in a Young Middle-Eastern Population. Eur. J. Endocrinol..

[88] Grimnes G., Figenschau Y., Almås B., Jorde R. (2011). Vitamin D, Insulin Secretion, Sensitivity, and Lipids Results from a Case-Control Study and a Randomized Controlled Trial Using Hyperglycemic Clamp Technique. Diabetes.

[89] Grineva E.N., Karonova T., Micheeva E., Belyaeva O., Nikitina I.L. (2013). Vitamin D Deficiency is a Risk Factor for Obesity and Diabetes Type 2 in Women at Late Reproductive Age. Aging.

[90] Ha C., Han T., Lee S., Cho J., Kang H. (2014). Association between Serum Vitamin D Status and Metabolic Syndrome in Korean Young Men. Epidemiology.

[91] Hao Y., Ma X., Shen Y., Ni J., Luo Y., Xiao Y., Bao Y., Jia W. (2014). Associations of Serum 25-Hydroxyvitamin D3 Levels with Visceral Adipose Tissue in Chinese Men with Normal Glucose Tolerance. PLoS ONE.

[92] Huang C., Chang H., Lu C., Tseng F., Lee L., Huang K. (2015). Vitamin D Status and Risk of Metabolic Syndrome among Non-diabetic Young Adults. Clin. Nutr..

[93] Kayaniyil S., Vieth R., Harris S.B., Retnakaran R., Knight J.A., Gerstein H.C., Perkins B.A., Zinman B., Hanley A.J. (2011). Association of 25(OH)D and PTH with Metabolic Syndrome and Its Traditional and Nontraditional Components. J. Clin. Endocrinol. Metab..

[94] Li H.W.R., Brereton R.E., Anderson R.A., Wallace A.M., Ho C.K.M. (2011). Vitamin D Deficiency is Common and Associated with Metabolic Risk Factors in Patients with Polycystic Ovary Syndrome. Metab. Clin. Exp..

[95] Liu E., Meigs J.B., Pittas A.G., McKeown N.M., Economos C.D., Booth S.L., Jacques P.F. (2009). Plasma 25-Hydroxyvitamin D Is Associated with Markers of the Insulin Resistant Phenotype in Nondiabetic Adults. J. Nutr..

[96] Lu L., Yu Z., Pan A., Hu F.B., Franco O.H., Li H., Li X., Yang X., Chen Y., Lin X. (2009). Plasma 25-hydroxyvitamin D Concentration and Metabolic Syndrome among Middle-aged and Elderly Chinese Individuals. Diabetes Care.

[97] Moore A., Hochner H., Sitlani C.M., Williams M.A., Hoofnagle A.N., de Boer I.H., Kestenbaum B., Siscovic D.S., Friedlander Y., Enquobahrie D.A. (2015). Plasma Vitamin D is Associated with Fasting Insulin and Homeostatic Model Assessment of Insulin Resistance in Young Adult Males, but Not Females, of the Jerusalem Perinatal Study. Public Health Nutr..

[98] Muscogiuri G., Sorice G.P., Prioletta A., Policola C., Casa S.D., Pontecorvi A., Giaccari A. (2010). 25-Hydroxyvitamin D Concentration Correlates with Insulin-Sensitivity and BMI in Obesity. Obesity.

[99] O’Hartaigh B., Thomas G.N., Silbernagel G.N., Bosch J.A., Pilz S., Loerbroks A., Kleber M.E., Grammer T.B., Bohm B.O., Marz W. (2013). Association of 25-hydroxyvitamin D with Type 2 Diabetes among Patients Undergoing Coronary Angiography: Cross-sectional Findings from the LUdwigshafen Risk and Cardiovascular Health (LURIC) Study. Clin. Endocrinol..

[100] Pham N.M., Akter S., Kurotani K., Nanri A., Sato M., Hayabuchi H., Yasuda K., Mizoue T. (2012). Serum 25-hydroxyvitamin D and Markers of Insulin Resistance in a Japanese Working Population. Eur. J. Clin. Nutr..

[101] Sabio J.M., Vargas-Hitos J.A., Martinez-Bordonado J., Navarrete-Navarrete N., Diaz-Chamorro A., Olvera-Porcel C., Zamora M., Jiménez-Alonso J. (2015). Association between Low 25-hydroxyvitamin D, Insulin Resistance and Arterial Stiffness in Nondiabetic Women with Systemic Lupus Erythematosus. Lupus.

[102] Sheth J.J., Shah A., Sheth F.J., Trivedi S., Lele M., Shah N., Thakor P., Vaidya R. (2015). Does Vitamin D Play a Significant Role in Type 2 Diabetes?. BMC Endocr. Disord..

[103] Sun X., Cao Z., Tanisawa K., Ito T., Oshima S., Higuchi M. (2014). The Relationship between Serum 25-Hydroxyvitamin D Concentration, Cardiorespiratory Fitness, and Insulin Resistance in Japanese Men. Nutrients.

[104] Tao M., Zhang Z., Yao-hua K.E., Jin-wei H.E., Wen-zhen F.U., Chang-qing Z., Zhen-lin Z. (2013). Association of Serum 25-hydroxyvitamin D with Insulin Resistance and β-cell Function in a Healthy Chinese Female Population. Acta Pharmacol. Sin..

[105] Tosunbayraktar G., Bas M., Kut A., Buyukkaragoz A.H. (2015). Low Serum 25(OH)D Levels are Assocıated to Higher BMI and Metabolic Syndrome Parameters in Adults Subjects in Turkey. Afr. Health Sci..

[106] Yin X., Sun Q., Zhang X., Lu Y., Sun C., Cui Y., Wang S. (2012). Serum 25(OH)D is Inversely Associated with Metabolic Syndrome Risk Profile among Urban Middle-aged Chinese Population. Nutr. J..

[107] Calvo-Romero J.M., Ramiro-Lozano J.M. (2015). Vitamin D Levels in Patients with Type 2 Diabetes Mellitus. J. Investig. Med..

[108] Cai X., Hu Z., Chen L., Han X., Ji L. (2014). Analysis of the Associations between Vitamin D and Albuminuria or Beta-Cell Function in Chinese Type 2 Diabetes. BioMed Res..

[109] Gao Y., Wu X., Fu Q., Li Y., Yang T., Tang W. (2015). The Relationship between Serum 25-hydroxyvitamin D and Insulin Sensitivity and Beta Cell Function in Newly Diagnosed Type 2 Diabetes. J. Diabetes Res..

[110] Rosen C.J., Adams J.S., Bikle D.D., Black D.M., Demay M.B., Manson J.E., Murad M.H., Kovacs C.S. (2012). The Nonskeletal Effects of Vitamin D: An Endocrine Society Scientific Statement. Endocr. Rev..

[111] Palomer X., González-Clemente J.M., Blanco-Vaca F., Mauricio D. (2008). Role of Vitamin D in the Pathogenesis of Type 2 Diabetes Mellitus. Diabetes Obes. Metab..

[112] Stein E.M., Strain G., Sinha N., Ortiz D., Pomp A., Dakin G., McMahon D.J., Bockman R., Silverberg S.J. (2009). Vitamin D Insufficiency Prior to Bariatric Surgery: Risk Factors and a Pilot Treatment Study. Clin. Endocrinol..

[113] Wamberg L., Cullberg K.B., Rejnmark L., Richelsen B., Pedersen S.B. (2013). Investigations of the Anti-inflammatory Effects of Vitamin D in Adipose Tissue: Results from an in vitro Study and a Randomized Controlled Trial. Horm. Metab. Res..

[114] Xu H., Barnes G.T., Yang Q. (2003). Chronic Inflammation in Fat Plays a Crucial Role in the Development of Obesity-related Insulin Resistance. J. Clin. Investig..

[115] Sasso F.C., Pafundi P.C., Marfella R., Calabrò P., Piscione F., Furbatto F., Esposito G., Galiero R., Gragnano F., Rinaldi L. (2019). Adiponectin and Insulin Resistance are Related to Restenosis and Overall New PCI in Subjects with Normal Glucose Tolerance: The Prospective AIRE Study. Cardiovasc. Diabetol..

[116] Mathieu C., Van Etten E., Gysemans C. (2001). In vitro and in vivo Analysis of the Immune System of Vitamin D Receptor Knockout Mice. J. Bone Miner. Res..

[117] Healy K.D., Frahm M.A., DeLuca H.F. (2005). 1,25-Dihydroxyvitamin D3 Up-regulates the Renal Vitamin D Receptor through Indirect Gene Activation and Receptor Stabilization. Arch. Biochem. Biophys..

[118] Cade C., Norman A.W. (1987). Rapid Normalization/Stimulation by 1,25-dihydroxyvitamin D3 of Insulin Secretion and Glucose Tolerance in the Vitamin D-deficient Rat. Endocrinology.

[119] Rafiq S., Jeppesen P.B. (2018). Is Hypovitaminosis D Related to Incidence of Type 2 Diabetes and High Fasting Glucose Level in Healthy Subjects: A Systematic Review and Meta-analysis of Observational Studies. Nutrients.

[120] Alkharfy K.M., Al-Daghri N.M., Yakout S.M. (2012). Calcitriol Attenuates Weight-related Systemic Inflammation and Ultrastructural Changes of the Liver in a Rodent Model. Basic Clin. Pharmacol. Toxicol..

[121] Barnard W.F., Saxena V.K., Wenny B.N., DeLuisi J.J. (2003). Daily Surface UV Exposure and Its Relationship to Surface Pollutant Measurements. J. Air Waste Manag. Assoc..

[122] Elminir H.K. (2007). Sensitivity of Ultraviolet Solar Radiation to Anthropogenic Air Pollutants and Weather Conditions. Atmos. Res..

[123] Alam U., Arul-Devah V., Javed S. (2016). Vitamin D and Diabetic Complications: True or False Prophet?. Diabetes Ther..

[124] Kaul K., Hodgkinson A., Tarr J.M., Kohner E.M., Chibber R. (2010). Is Inflammation a Common Retinal-renal-nerve Pathogenic Link in Diabetes?. Curr. Diabetes Rev..

[125] Burton J.M., Kimball S., Vieth R. (2010). A Phase I/II Dose-escalation Trial of Vitamin D3 and Calcium in Multiple Sclerosis. Neurology.

[126] Holmøy T., Moen S.M. (2010). Assessing Vitamin D in the Central Nervous System. Acta Neurol. Scand. Suppl..

[127] Simon K.C., Munger K.L., Ascherio A. (2012). Vitamin D and Multiple Sclerosis: Epidemiology, Immunology, and Genetics. Curr. Opin. Neurol..

[128] Fex S.A., Dahlin L.B. (2013). Repair of the Peripheral Nerve—Remyelination That Works. Brain Sci..

[129] Neveu I., Naveilhan P., Jehan F. (1994). 1,25-Dihydroxyvitamin D3 Regulates the Synthesis of Nerve Growth Factor in Primary Cultures of Glial Cells. Brain Res. Mol. Brain Res..

[130] Ito S., Ohtsuki S., Nezu Y., Koitabashi Y., Murata S., Terasaki T. (2011). 1,25-Dihydroxyvitamin D3 Enhances Cerebral Clearance of Human Amyloid-b Peptide (1-40) from Mouse Brain Across the Blood-brain Barrier. Fluids Barriers CNS.

[131] Generini S., Tuveri M.A., Matucci Cerinic M., Mastinu F., Manni L., Aloe L. (2004). Topical Application of Nerve Growth Factor in Human Diabetic Foot Ulcers. A Study of Three Cases. Exp. Clin. Endocrinol. Diabetes.

